# Measuring functional disability in children with developmental disorders in low-resource settings: validation of Developmental Disorders-Children Disability Assessment Schedule (DD-CDAS) in rural Pakistan

**DOI:** 10.1017/gmh.2020.10

**Published:** 2020-07-13

**Authors:** Syed Usman Hamdani, Zill-e Huma, Lawrence Wissow, Atif Rahman, Melissa Gladstone

**Affiliations:** 1 University of Liverpool, Liverpool, UK; 2 Human Development Research Foundation, Islamabad, Pakistan; 3 University of Washington, Seattle, USA

**Keywords:** Autism, childhood disability, Developmental Disabilities Children's Global Assessment Scale (DD-CGAS), developmental disorders/disabilities, Developmental Disorders-Children Disability Assessment Schedule (DD-CDAS), functional disability, intellectual disability, International Classification of Functioning-Children and Youth (ICF-CY), low-resource settings, non-specialists, WHO Disability Assessment Schedule (WHODAS 2.0), DD-CDAS, Developmental Disorders-Children Disability Assessment Schedule, ICF, International Classification of Functioning, ICF-CY, International Classification of Functioning-Children and Youth, TQS, Ten Questions Screen, WHO mhGAP, World Health Organization Mental Health Gap Action Programme, CGAS, Children-Global Assessment of Functioning, DD-CGAS, Developmental Disorders Children-Global Assessment of Functioning, VABS, Vineland Adaptive Behavior Scales, CFA, Confirmatory Factor Analysis, RMSEA, Root Mean Square Error of Approximation, TLI, Tucker–Lewis Index, CFI, Comparative Fit Index, IFI, Incremental Fit Index, AMOS, Analysis of Moment Structure, ICC, Interclass Correlation, SDGs, Sustainable Development Goals, LHWs, Lady Health Workers.

## Abstract

**Background:**

Developmental disorders (DDs) in children are a priority condition and guidelines have been developed for their management within low-resource community settings. However, a key obstacle is lack of open access, reliable and valid tools that lay health workers can use to evaluate the impact of such programmes on child outcomes. We adapted and validated the World Health Organization's Disability Assessment Schedule for children (WHODAS-Child), a lay health worker-administered functioning-related tool, for children with DDs in Pakistan.

**Methods:**

Lay health workers administered a version of the WHODAS-Child to parents of children with DDs (*N* = 400) and without DDs (*N* = 400), aged 2–12 years, after it was adapted using qualitative study. Factor analysis, validity, reliability and sensitivity to change analyses were conducted to evaluate the psychometric properties of the adapted outcome measure.

**Results:**

Among 800 children, 58% of children were male [mean (s.d.) age 6.68 (s.d. = 2.89)]. Confirmatory Factor Analysis showed a robust factor structure [χ^2^/df 2.86, RMSEA 0.068 (90% CI 0.064–0.073); Tucker–Lewis Index (TLI) 0.92; Comparative Fit Index (CFI) 0.93; Incremental Fit Index (IFI) 0.93]. The tool demonstrated high internal consistency (*α* 0.82–0.94), test–retest [Intra-class Correlation Coefficient (ICC) 0.71–0.98] and inter-data collector (ICC 0.97–0.99) reliabilities; good criterion (*r* −0.71), convergent (*r* −0.35 to 0.71) and discriminative [M (s.d.) 52.00 (s.d. = 21.97) *v*. 2.14 (s.d. = 4.00); 95% CI −52.05 to −47.67] validities; and adequate sensitivity to change over time (ES 0.19–0.23).

**Conclusions:**

The lay health worker administrated version of adapted WHODAS-Child is a reliable, valid and sensitive-to-change measure of functional disability in children aged 2–12 years with DDs in rural community settings of Pakistan.

## Background

In health care sites in low-resource settings, providers frequently encounter children with physical and cognitive disabilities. Rapid unplanned urbanization, inequitable distribution of resources, fragile health systems, poor obstetric practices, malnutrition and poverty continue to increase the risk of poor child development and child neurodisability in low-resource settings globally (Groce *et al*., 2014). The focus of the new United Nations (UN) global strategy for women, children and adolescents is to move beyond survival, to ensuring health and wellbeing for everyone and on providing enabling environments (Kuruvilla *et al*., 2016). Measuring functioning of an individual in various life domains, in different contexts, and for various states of health and disease is a critical piece of information to inform individualized intervention plans, programmes and health policies (Murray, 2002). To materialize this vision, standardized and uniform measurement frameworks such as the ICF (International Classification of Functioning, Disability and Health) (WHO, 2001) and tools based on the ICF such as the WHO Disability Assessment Schedule (WHODAS-2.0) for adults have been developed and validated (Üstün *et al*., 2010). WHODAS-2.0 is an open access tool that has been used cross-culturally for various health conditions to provide important health policy information through population surveys (Üstün *et al*., 2001; Buist-Bouwman *et al*., 2008; Sousa *et al*., 2009) and health registries (Gallagher and Mulvany, 2004). It has also been utilized in formulating and monitoring individualized treatment plans and evaluating effectiveness and cost-effectiveness of public health interventions for adults (Chopra *et al*., 2004; McKibbin *et al*., 2004; Chisolm *et al*., 2005; Perini *et al*., 2006; Soberg *et al*., 2007; Baron *et al*., 2008; Hudson *et al*., 2008; Schlote *et al*., 2008; Federici *et al*., 2009).

Many of the child and youth disabilities encountered by health care workers and researchers in low-resource settings fall into the category of neurodevelopmental disorders (Olusanya *et al*., 2018). The World Health Organization's Mental Health Gap Action Programme (WHO mhGAP) intervention guide (mhGAP-IG) uses the related term ‘Developmental Disorder’ (DD) to define ‘an impairment or delay in functions related to central nervous system maturation impacting multiple developmental domains’ (WHO, 2017). Thus, developmental disorder is an umbrella term covering a range of conditions, including intellectual disability as well as autism spectrum disorders, with the common factor being intellectual and adaptive functioning deficits. The American Psychiatric Association uses the term neurodevelopmental disorder which is defined as, ‘a group of congenital or acquired long-term conditions that are attributed to impairment of the brain and/or neuromuscular system and create functional limitations’ (American Psychiatric Association, 2013). The symptoms of neurodevelopmental disorders may occur in combination with other disorders and the symptoms' severity and complexity may vary over time. Neurodevelopmental disorders may cause difficulties in ‘movement, cognition, hearing and vision, communication, emotion and behaviour’. Common to all neurodevelopmental disorders is the key characteristic of (a) origin in early childhood and (b) impact on multiple developmental domains (motor, cognitive, behavioural and communication) (WHO, 2017). However, no tools like the WHODAS-2.0 exist for children with neurodevelopmental and/or developmental disorders that are open source, simple to administer, and valid over a range of child development domains, age groups and socio-cultural settings.

Traditional assessment of childhood developmental disorders either involves screening by using brief, valid questionnaires such as Ten Questions Screening Tool (Durkin *et al*., 1995*b*) to identify children with or without disability or detailed developmental assessment using standardized tools by a multidisciplinary team of experts for diagnosis and evaluation of adaptive functioning in children. The brief screening tools are reliable and valid for screening purpose only, i.e. they fail to measure the impact of child disability on child functioning. The standard tools for assessment are often copyrighted, need extensive training and supervision for administration, scoring and interpretation, and thus are not available in low-resource settings or practical in pragmatic research.

One reason why more simple tools for children can be difficult to develop is that children's functioning is so highly context and culturally specific (Scorza *et al*., 2013). The WHO's ICF-CY (International Classification of Functioning-Child and Youth version) provides a common terminology for identifying functional problems in children and adolescents, including bodily functioning, activity limitations and participation restrictions (Scorza *et al*., 2013), but there has been limited progress towards development and validation of measurement tools which are grounded in this potentially trans-cultural framework. One group has developed and validated a youth version of WHODAS 2.0 for adolescents (aged 10–17) with mental health problems in rural Rwanda (Scorza *et al*., 2013) and named it WHODAS-Child. The tool demonstrated good internal consistency (*α* = 0.83) and reliability in measuring functional impairment among adolescents with socio-emotional problems, but there remains a need for validation of a tool suitable for younger children with developmental disorders.

In this paper, we describe the validation of WHODAS-Child (Scorza *et al*., 2013) for use with children with developmental disorders in Pakistan. We followed the WHO mhGAP-IG definition of development disorders, ‘development disorder is an umbrella term covering disorders such as intellectual disability as well as autism spectrum disorders. These disorders usually have a childhood onset, impairment or delay in functions related to central nervous system maturation, and a steady course rather than the remissions and relapses that tend to characterize many other mental disorders’ (WHO, 2017). This paper describes the results of factor analysis, reliability, validity and sensitivity to change analyses of the adapted WHODAS-Child in rural Pakistan.

## Methods

### Study settings

We conducted the study in the rural sub-district (Gujar Khan) of Rawalpindi, Pakistan (estimated population of one million). In Pakistan, the smallest administrative unit in a sub-district is a Union Council (UC). Each UC has about 12–15 villages with a population of approximately 30 000–50 000. Each UC has a Primary Health Care (PHC) facility called a Basic Health Unit (BHU). The BHU has a work force of a primary care physician or a medical technician, a Lady Health Visitor (LHV), a vaccinator and a set of Lady Health Workers (LHWs). The study was conducted in 30 UCs of sub-district Gujar Khan. The infant mortality rate in Rawalpindi has been estimated to be 55 per thousand live births, the under-5 mortality rate is 82 per thousand live births and the percentage of underweight children below 5 years of age is 25% (Bureau of Statistics & UNICEF Pakistan, 2003). In a previous study, 34.2% of children in Rawalpindi district were screened positive on the Ten Questions Screen (TQS) for childhood developmental difficulties (Mirza *et al*., 2008).

### Sampling

The data for this validation study were collected as a part of an implementation research trial embedded within the scaled-up implementation of WHO mhGAP-based Parent Skills Training (PST) programme in rural Pakistan (Hamdani *et al*., 2017). The host organization has a database of about 3000 families and children with developmental disorders as a part of the host organization's service delivery to the community (Hamdani *et al*., 2015). For the purpose of the cluster randomized controlled trial (cRCT), a sample size of 540 parent–child dyads from 30 clusters (18 parent–child dyads per cluster) was required to evaluate the impact of PST programme (Hamdani *et al*., 2017). To ensure the representativeness of the study sample, evaluation zones were created within each UC. These evaluation zones were made by randomly selecting one LHW out of the total 15–20 LHWs serving in a particular UC. The random selection was carried out by an independent researcher using a simple random table. If the required number of dyads was not completed from one LHW catchment area, the adjacent LHW catchment area was included to reach the required sample size from each UC. This patch of contiguous area/catchment areas of LHWs within that UC served as the impact evaluation zone for the trial-related outcomes. This sampling process ensured that 540 parent–child dyads (18 parent–child dyads per cluster) are randomly selected from 3000 families and children participating in the scaled-up implementation of programme. For the purpose of validation study, a sub-set of the data consisting of 400 parent–child dyads was randomly selected using Statistical Package for Social Sciences (SPSS).

### Participants

The target population for this study was the parents/caregivers of children with developmental disorders aged 2–12 years. The study sample (*N* = 800) involved 400 parents of children with developmental disorders and 400 parents of age- and gender-matched children without developmental disorders from the same community.

Our approach to identify children with development disorders in rural community settings is informed by a similar study in rural India by Singhi *et al*. (2007). In phase 1, TQS was administered to parents of children aged between 2 and 12 years. In phase 2, all children who screened positive were clinically evaluated in detail following the mhGAP guidelines by a trained psychologist.

### Children with developmental disorders

#### Inclusion criteria

- Children who screened positive on any of the TQS questionnaire items # 1, 4, 5, 7, 8, 9, 10 for developmental delay (Durkin *et al*., 1995*a*).
- Identified as developmental disorder in the child, according to clinical assessment (history and clinical examination for developmental delay in motor, communication, social, cognitive, daily living skills domains according to WHO mhGAP developmental disorders guidelines for clinical assessment in primary healthcare settings).

#### Exclusion criteria

Exclusion criteria include

- Deafness or blindness in the child or caregiver.
- Primary caregiver not available or unwilling to participate in the study.
- Primary caregiver unable to speak or understand the Urdu language.

### Children without developmental disorders

#### Inclusion criteria

- Primary caregiver of children aged 2–12 years, who screened negative for developmental disorders using TQS.
- Residing in the study area.

#### Exclusion criterion

- Inability to speak or understand the Urdu language.

The Nquery Advisor (Elashoff, 2014) was used to estimate the sample size for test–retest analysis, inter-rater reliability analysis, discriminative reliability and convergent validity. To determine the sufficiency of sample size to estimate a fit for the model, we used the ratio of 10 participants (N) for 1 parameter (p). This is consistent with sample size calculations for Confirmatory Factor Analysis (CFA) in the Rwanda study where with a sample size of 367, the data set met minimum sample guidelines for the CFA (MacCallum *et al*., 1999).

### Data collection procedures

The current study was embedded within a scaled-up implementation of a PST programme, based on guidance from WHO's mhGAP-IG (WHO, 2017) and using core contents (‘key messages’ and strategies) of the WHO Caregivers Skills Training (CST) programme (Salomone *et al*., 2019). Data collection took place over a period of 6 months (Hamdani *et al*., 2017). To overcome the barrier of illiteracy, all instruments were administered by trained interviewers to the parents/caregivers. Non-specialist health care workers were trained prior to data collection and those with good inter-data collector reliability scores ICC >0.80 (Koo and Li, 2016) were selected to collect data. Data were collected electronically using handheld devices on an android-based application named Open Data Kit (ODK)^1^† and checked periodically for consistency and completeness.

Ethics approval for the study was obtained from the Ethics Review Committee of the Human Development Research Foundation, Islamabad, Pakistan. Parental written informed consent was obtained from all participants, either by written signature or by finger prints depending upon literacy level of the parents/caregivers. All participating children had access to primary healthcare centres and specialist child mental health unit at the Institute of Psychiatry, WHO Collaborating Centre for mental health research and training, Rawalpindi (WHO-CC) (the tertiary mental health care facility in the study sub-district).

### Translation and adaptation of WHODAS-Child for children with developmental disorders (*henceforth called* Developmental Disorders-Children Disability Assessment Schedule – DD-CDAS)

We used a mixed-methods approach to translate and culturally adapt the WHODAS-Child into Urdu for administration by an interviewer. Interviews were conducted with parents of children with and without developmental disorders to capture local norms of age-appropriate healthy functioning in children through free list interviews (Van Ommeren *et al*., 1999). We then made general adaptations such as (a) rephrasing items to target parents/caregivers of children as respondents, (b) making each item more comprehensible for parents with a common stem added to each item and (c) providing culturally specific examples of child functioning based upon the free list interviews. Details of the qualitative study with the free list interviews as well as the translation and adaptation process are reported elsewhere (Hamdani *et al*., forthcoming). The initial working version of the adapted WHODAS-Child for children with developmental disorders (DD-CDAS), prior to further validation, consisted of 36 items on functioning and disability covering six domains with a recall period of 30 days. The distribution of items in the six domains was as follows: understanding and communication (six items), getting around (five items), self-care (four items), getting along with others (five items), life activities (four items related to household activities and five items for work/school) and participation in society (seven items). The 36 items were rated on a five-point scale where 1 = *none*, 2 = *mild*, 3 = *moderate*, 4 = *severe*, 5 = *extreme/cannot do*. Using the standard scoring algorithm from WHODAS-Child, school-related items were not rated for children who were not attending the school and life activities domain and total disability scores were computed from the remaining items (Üstün *et al*., 2010). Domain and total raw scores were transformed into a range from 0 to 100. A global disability score was computed from all 36 items. A higher score indicates greater disability or difficulty in functioning. DD-CDAS items along with their corresponding ICF-CY codes are given as an Additional file A.

**Criterion validity** was assessed by correlating DD-CDAS global disability score with clinician-rated Developmental Disabilities Children's Global Assessment Scale (DD-CGAS) scores (Shaffer *et al*., 1983). The DD-CGAS rating is a global rating of functioning in children based on all available sources of information and across all domains of functioning, including self-care, communication, social behaviour and school/academic functioning (Wagner *et al*., 2007; Olsson and Bölte, 2014; White *et al*., 2014). A high inverse correlation was expected as the DD-CDAS measures the construct of difficulty in functioning whereas DD-CGAS is a measure of global functioning.

**Convergent validity:** We used the Vineland Adaptive Behaviour Composite (VABS-II-ABC score) (Sparrow *et al*., 2005) and domain scores to assess the convergent validity of DD-CDAS. The VABS-II-ABC score measures the adaptive functioning of individuals from birth to 90 years of age. VABS-II consists of five domains with each having 2–3 sub-domains. The primary domains are communication, daily living skills, socialization and motor skills. A total sum of all domains produces an Adaptive Behaviour Composite score. A higher score indicates better functioning. An adapted version of VABS-II for children with developmental disorders was used in the present study (Rahman *et al*., 2016). Data to assess the convergent validity was obtained from a smaller sub-sample (*N* = 68).

We did a correlation analysis between the pro-social domain scores of Strengths and Difficulties Questionnaire (SDQ) (Goodman, 1997) and functional disability of DD-CDAS in children with developmental disorders. The pro-social domain of SDQ measures how much a child is considerate of other people's feelings; shares readily; helpful if someone is hurt. The higher the score, the more pro-social a child is. We used the parent-rated version of the tool which has been validated in a similar population (Maselko *et al*., 2016).

**Discriminative validity** of DD-CDAS was assessed by comparing the total score and domains score of age- and gender-matched children with developmental disorders with the control sample of children without developmental disorders. The total difficulty scores of children without developmental disorders were expected to be better (lower) than children with developmental disorders.

**Subgroup analysis** was conducted to evaluate DD-CDAS ability to differentiate among members of the sample with different developmental disorders. Descriptive statistics were calculated to compare domains and global disability scores across groups.

**Reliability** was assessed through measuring internal consistency, test–retest (reproducibility) and inter-data collector reliability (where only the data collector was changed, not the respondent). Internal consistency was evaluated with the Cronbach's *α* coefficients. To assess test–retest, a sub-sample of participants was identified and assessed at 2-week intervals by the same rater. As the sample consisted of children with developmental disorders, their clinical severity was not expected to change over a period of 2 weeks. Inter-data collector reliability was assessed by administering the DD-CDAS to the same caregivers by two different non-specialist health care providers. Concordance in the scores was estimated by calculating the One-way Random Intra-class Correlation Coefficient (ICC).

**Sensitivity to change:** The responsiveness of DD-CDAS was evaluated by administering it 6 months after pre-test. The study sample consisted of caregivers of children with developmental disorders, who received training in the PST programme (Hamdani *et al*., 2017).

**CFA of DD-CDAS:** CFA was performed to assess the hypothesized six-domain structure of the DD-CDAS, i.e. understanding and communication (cognition), getting around (mobility), self-care, getting along with people, life activities and participation in society. Goodness-of-fit was measured by the Root Mean Square Error of Approximation (RMSEA, adequate if below 0.08), Comparative Fit Index (CFI), Incremental Fit Index (IFI) and Tucker–Lewis Index (TLI), which are recommended to be over 0.90 (Hooper *et al*., 2008). The relative/normed χ^2^/df (Wheaton *et al*., 1977) was used to minimize the impact of sample size on model fit (Hooper *et al*., 2008). CFA was conducted using AMOS version 21.0 with maximum likelihood estimation. Missing values were considered missing at random and were handled by using maximum likelihood estimation due to the very small number of missing values in the study data.

## Results

Descriptive statistics for DD-CDAS global disability scores and domain scores were examined for children with and without developmental disorders.

### Sample characteristics

Table 1 shows sample characteristics. In the developmental disorders sample, the majority of children were male (62%); 37% of the participants were aged between 2 and 5 years, 33% were aged between 6 and 8 years and 30% were aged between 9 and 12 years. Among them, most of the children were not attending schools (69%). In 96% of cases, mothers were the primary caregiver. There was a significant difference between both groups in terms of gender distribution, family structure, history of developmental disorder in family and number of children attending schools. Only 1% sample of the children with developmental disorders were attending special school.

**Table 1.** Demographic characteristics of study participants (*N* = 800)

Variables	Children with developmental disorders (*n* = 400)	Children without developmental disorders (*n* = 400)	Total (*N* = 800)	*p*
Gender				
Male	248 (62%)	217 (54%)	465 (58%)	0.01
Female	152 (38%)	183 (45%)	335 (42%)	
Age				
2–5 years	149 (37%)	156(39%)	305 (38%)	0.85
6–8 years	132 (33%)	131 (32%)	263 (32.9%)	
9–12 years	119 (30%)	113 (28%)	232 (29%)	
Children attending school				
No	279 (69%)	80 (20%)	359 (44.9%)	0.00
Yes	121 (30%)	320 (80%)	441 (55.1%)	
Family structure				
Nuclear	179 (44.8%)	133 (33%)	312 (39%)	0.00
Joint/extended	208 (52%)	244 (61%)	452 (56.5%)	
Multiple households	13 (3%)	23 (5.8%)	36 (4.5%)	
Primary caregiver of child (study informant)				
Mother	385 (96%)	387 (96%)	772 (96.5%)	0.20
Sister	3 (1%)	1 (0.3%)	4 (0.5%)	
Paternal aunt	6 (2%)	3 (1%)	9 (1.1%)	
Maternal aunt	2 (1%)	0(0)	2 (0.3%)	
Grand mother	4 (1%)	9 (2%)	13 (1.6%)	
Family history of developmental disorders				
No	280 (70%)	355 (88.8%)	635 (79.4%)	0.00
Yes	120 (30%)	45 (11.3%)	165 (20.6%)	
Results of screening on TQS				
Cognitive difficulties	246 (61.5)			
Motor difficulties	30 (7.5)			
Cognitive difficulties with motor difficulties	110 (27.5)			
Communication difficulties	14 (3.5)			

TQS , Ten Questions Screening.

### Descriptive statistics

**Fig. 1.**
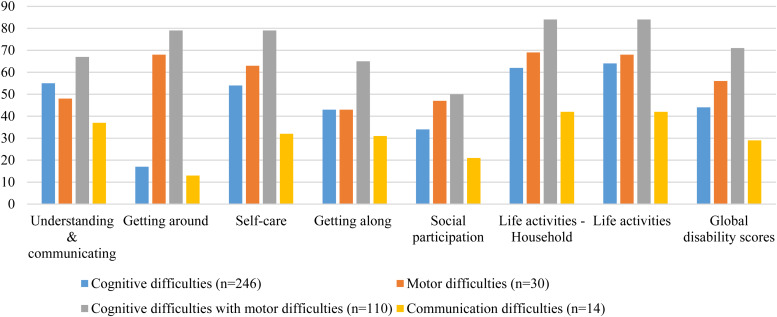
Developmental Disorders-Children Disability Assessment Schedule (DD-CDAS) domain profile by sub-group (N=400) (*N* = 800).

**Fig. 2.**
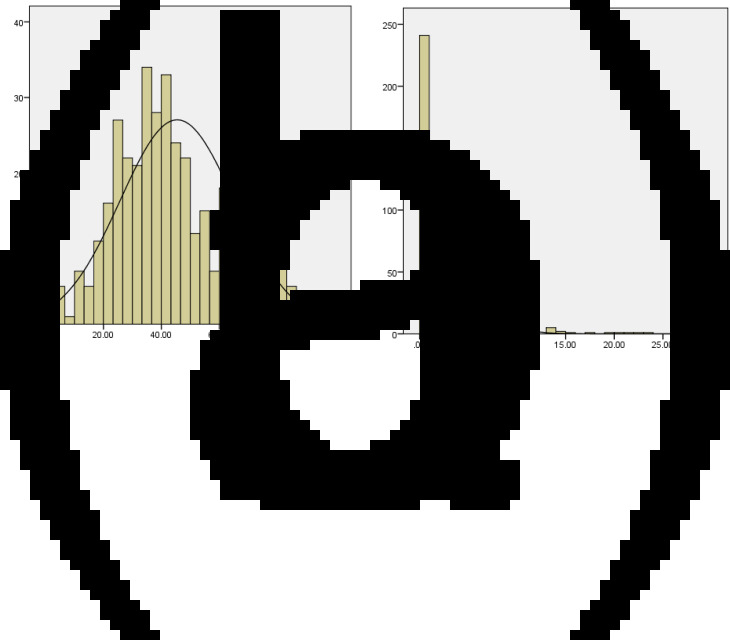
Distribution curve for ‘global disability score’ of DD-CDAS for children with and without developmental disorders (*N* = 800).

The average interview time based on an informal estimation of data collectors to administer DD-CDAS was 20 minutes. The distribution of DD-CDAS scores for children with and without developmental disorders is reported in Table 2 and distribution of DD-CDAS scores for children with developmental disorders in Fig. 1. The mean global disability score for children with developmental disorders was 52.00 (s.d. = 21.98) ranging from 4.17 to 97.92, while for children without developmental disorders, the mean global disability score was 2.14 (s.d. = 4.00) ranging from 0 to 26.06. In children with developmental disabilities, no significant differences were observed between girls and boys on global disability score [51.87 (s.d. = 22.06) *v*. 52.21 (s.d. = 21.88); 95% CI −4.80 to 4.10]. Similarly, no significant differences were observed in sub-domain scores between boys and girls. The normal distribution curves with a histogram for ‘global disability score’ of DD-CDAS for children with and without developmental disorders are mentioned in Fig. 2.

**Table 2.** DD-CDAS domains profile by sub-group (*N* = 800)

Children with and without developmental disorders conditions	Understanding and communicating *M* (s.d.)	Getting around *M (*s.d.*)*	Self-care *M (*s.d.*)*	Getting along *M (*s.d.*)*	Social participation *M (*s.d.*)*	^a^Life activities – household *M (*s.d.*)*	^a^Life activities – school *M (*s.d.*)*	Life activities *M (*s.d.*)*	Global disability scores *M (*s.d.*)*	DD-CGAS *M (*s.d.*)*
Cognitive difficulties (*n* = 246)	55 (21.43)	17 (27.45)	54 (26.01)	43 (26.52)	34 (20.23)	62 (20.89)	63 (22.29)	64 (20.77)	44 (16.89)	47.58 (12.45)
Motor difficulties (*n* = 30)	48 (28.88)	68 (29.87)	63 (28.38)	43 (26.25)	47 (20.88)	69 (25.37)	41 (40.06)	68 (27.19)	56 (22.12)	50 (18.73)
Cognitive difficulties with motor difficulties (*n* = 110)	67 (25.60)	79 (31.42)	79 (31.42)	65 (23.97)	50 (19.52)	84 (22.20)	66 (21.66)	84 (21.84)	71 (20.03)	36.91 (16.66)
Communication difficulties (*n* = 14)	37 (22.32)	13 (23.58)	32 (24.86)	31 (26.76)	21 (22.14)	42 (25.29)	34 (25.35)	42 (24.47)	29 (19.30)	56.71 (9.76)
Children without developmental disorders (*n* = 400)	2 (6.15)	1 (3.53)	3 (7.90)	2 (6.17)	4 (8.98)	4 (10.78)	5 (11.81)	4 (10.01)	2 (4)	91.40 (5.33)

DD-CDAS, Developmental Disorders-Children Disability Assessment Schedule; DD-CGAS, Developmental Disabilities Children's Global Assessment Scale.

a Data of children with developmental disorders who attended the school (31%) is included in analysis only.

### Confirmatory Factor Analysis

**Fig. 3.**
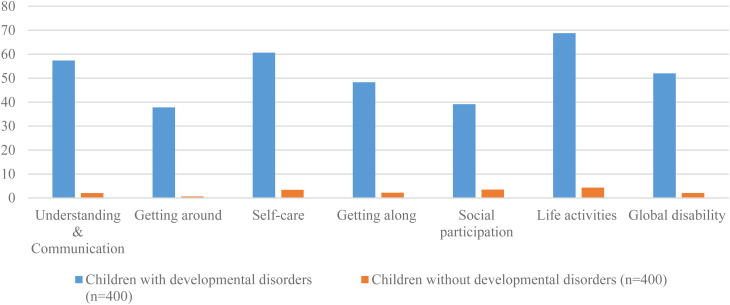
First-order Confirmatory Factor Analysis of the Developmental Disorders-Children Disability Assessment Schedule (DD-CDAS).

**Fig. 4.**
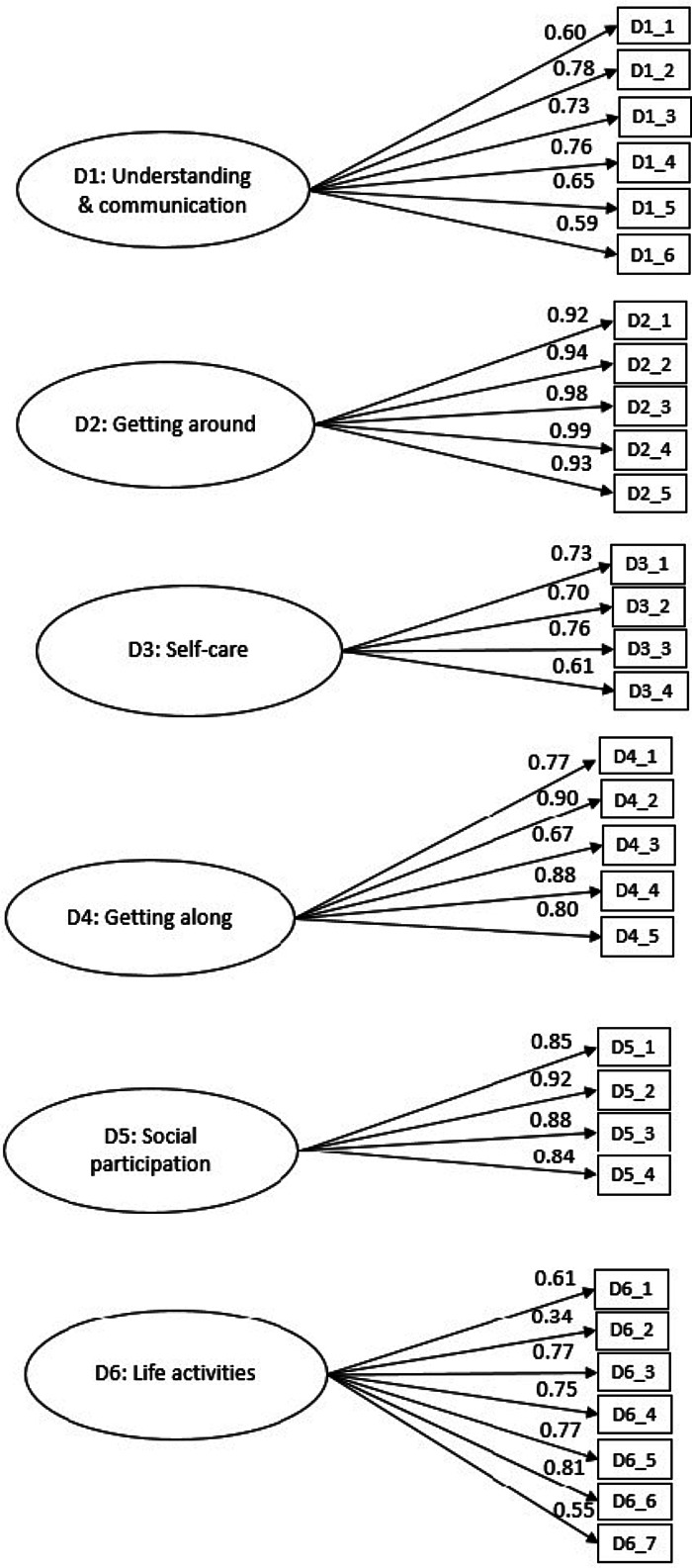
Second-order Confirmatory Factor Analysis of Developmental Disorders-Children Disability Assessment Schedule (DD-CDAS).

CFA was conducted on the data collected from caregivers of children with developmental disorders. As the majority of these children were not attending school, we conducted factor analysis (and all other analyses) on 31 items excluding five school-related items. CFA confirmed that the six-factor model structure of the DD-CDAS is consistent with the structure of the original WHODAS-Child. The values of the RMSEA [χ^2^/df 2.86, RMSEA 0.068 (90% CI 0.064–0.073), TLI 0.92, CFI 0.93 and IFI 0.93] (Fig. 3) indicated good fit for the six-factor model (Scorza *et al*., 2013), also indicating acceptable model fit (Hooper *et al*., 2008; Jackson *et al*., 2009) with all items loading to their respective domains. Only one item in the domain of participation in society, ‘*In your opinion, to what extent did your child not get opportunities to take part in social activities the way they ought to have been given to him/her?*’, had a factor loading of 0.34. Second-order CFA confirmed a two-level hierarchical structure, with one global disability factor feeding into the six domains [χ^2^/df 2.70, RMSEA 0.065 (90% CI 0.061–0.070), TLI 0.93, CFI 0.93 and IFI 0.93] (Fig. 4).

### Criterion validity

A high inverse correlation of (*r* = −0.70, *p* < 0.01) was observed between DD-CGAS and DD-CDAS, indicating that both scales measure similar construct in opposite ways; one measuring functioning and other measuring functioning impairment or disability.

### Convergent validity

We found moderate to high correlation coefficients between DD-CDAS in comparison with the Vineland Adaptive Behaviour Scale II (VABS II) (Table 3). The constructs of the DD-CDAS correlated well with similar constructs of VABS II. For example, the DD-CDAS constructs of ‘understanding and communication’ were well correlated with ‘daily living skills (−0.63) and communication (−0.60)’ of the VABS II and the DD-CDAS construct of ‘self-care’ was well correlated with ‘adaptive behaviour (−0.60) and daily living skills (−0.64)’ constructs of VABS II. Most of the other coefficients ranged between 0.43 and 0.77, except 0.35 between participation in society and socialization domains.

**Table 3.** Correlation coefficients between Vineland Adaptive Behaviour Scales II (VABS-II) and DD-CDAS (*N* = 68)

Vineland Adaptive Behaviour Scales II (VABS-II)					
DD-CDAS Domains	Adaptive behaviour (composite)	Socialization	Daily living skills	Communication	Motor skills
Understanding and communication	−0.53**	−0.57**	−0.63**	−0.60**	−0.67**
Getting along	−0.41**	−0.54**	−0.56**	−0.58**	−0.53**
Life activities	−0.52**	−0.43**	−0.58**	−0.53**	−0.63**
Getting around	−0.56**	−0.36**	−0.46**	−0.38**	−0.77**
Self-care	−0.60**	−0.52**	−0.64**	−0.53**	−0.77**
Social participation	−0.51**	−0.35**	−0.47**	−0.41**	−0.54**
Global disability	−0.62**	−0.54**	−0.65**	−0.59**	−0.78**

DD-CDAS, Developmental Disorders-Children Disability Assessment Schedule.

**p < 0.01.

Table 4 describes the correlation of DD-CDAS with the pro-social behaviour subscale of SDQ. The moderate negative correlation (*r* = −0.57, *p* < 0.01) between the two measures highlights that the children with DDs have difficulties with prosocial behaviour (Russell *et al*., 2013).

**Table 4.** Correlation analysis of DD-CDAS with pro-social construct of SDQ (*N* = 400)

Scale/subscale	Correlation
Global disability score	−0.57**
Pro-social behaviour score	–

DD-CDAS-DDs, Developmental Disorders-Children Disability Assessment Schedule; SDQ, Strengths and Difficulties Questionnaire.

**p < 0.01.

### Discriminative validity

We observed significant differences between children with and without developmental disorders on the DD-CDAS global disability mean score and the DD-CDAS domain subscales (Table 2) We found that the mean scores of children with developmental disorders were high on all domains of the DD-CDAS as compared to children without developmental disorders, reflecting that children with developmental disorders are significantly more likely to experience problems in managing life activities in comparison to children without developmental disorders.

### Internal consistency

The internal consistency between DD-CDAS items was high. Cronbach's *α* coefficients for the different DD-CDAS domains were as follows: understanding and communication (six items), 0.85; getting around (five items), 0.92; self-care (four items), 0.84; getting along with people (five items), 0.92; life activities – household (four items), 0.94; life activities – school (five items), 0.92; and participation in society (seven items), 0.82. Total internal consistency of the DD-CDAS was 0.91 for 36 items and 0.95 for 31 items excluding school-related items.

### Test–retest reliability

The DD-CDAS showed good test–rest reliability. The ICC ranged from 0.83 to 0.99 for domains, and 0.98 for overall score. All correlations were significant at 0.01 level.

### Inter-data collector reliability

The results of the two observations for the same case by two assessors were correlated to evaluate the inter-data collector reliability of disability assessment schedule. The ICCs for the inter-data collector reliability of DD-CDAS ranged from 0.97 to 0.99 (*p* < 0.01).

### Subgroup analysis

- Age groups (2–5, 6–8 and 9–12 years)

The mean global disability score in children aged 2–5 years was 55.23 (s.d. = 23.68), compared to 52.19 among children aged 6–8 years (s.d. = 22.02), while for children aged 9–12 years, the mean global disability score was 47.51 (s.d. = 18.81). There was a statistically significant difference among the three age groups on global disability score (*F* = 4.46, *p* = 0.021); and the getting around (*F* = 8.84, *p* < 0.021), self-care (*F* = 9.69, *p* = 0.01) domains of DD-CDAS as determined by one-way ANOVA (see Table 5).

**Table 5.** Age differences in DD-CDAS scores (*N* = 400)

DD-CDAS domains	2–5 years (*n* = 149)	6–8 years (*n* = 132)	9–12 years (*n* = 119)	*F*	*P*	95% CI	
	*M* (s.d.)	*M* (s.d.)	*M* (s.d.)			*LL*	*UL*
Global disability score	55.23 (23.68)	52.19 (22.02)	47.51 (18.81)	4.43	0.01*	49.83	54.15
Understanding and communication	57.29 (30)	57.89 (22.43)	56.65 (21.80)	0.08	0.91	55.02	59.68
Getting around	46.58 (41.61)	37.99 (41.48)	26.30 (34.88)	8.84	0.00*	33.82	41.27
Self-care	65.87 (27.02)	62.78 (27.17)	51.47 (29.39)	9.69	0.00*	57.84	63.43
Getting along	51.11 (30.16)	46.74 (28.07)	46.17 (26.24)	1.38	0.28	45.49	51.07
Life activities	68.59 (26.77)	69.43 (23.52)	68.13 (20.92)	0.09	0.90	66.39	71.11
Participation in society	41.91 (21.91)	38.31 (22.68)	36.37 (19.61)	2.51	0.08	37.03	41.28

*DD-CDAS, Developmental Disorders-Children Disability Assessment Schedule.

**Fig. 5.**
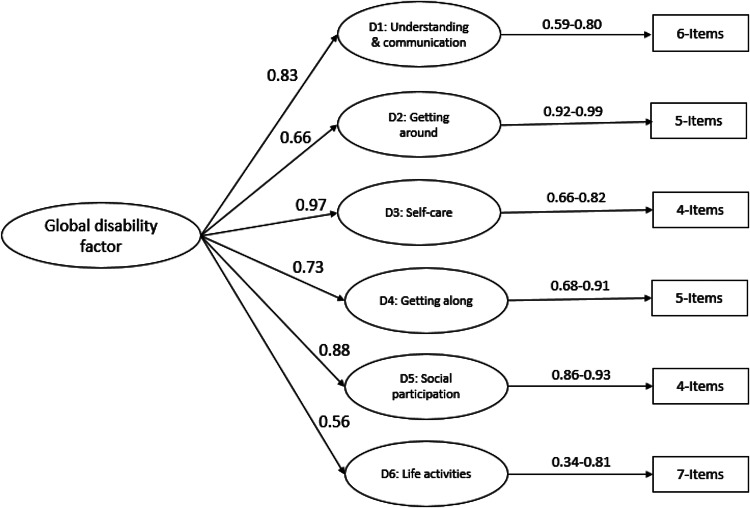
DD-CDAS domain profile by sub-group (*N* = 400).

Figure 5 shows that DD-CDAS domain scores for children with specific developmental difficulties were in line with their disabilities. Children with motor difficulties (*n* = 30) had higher disability scores on ‘getting around’ [68.33 (s.d.  29.86)] and ‘life activities’ [67.56 (s.d. = 27.18)] domains, while children with cognitive impairment with motor difficulties (*n* = 110) exhibited the highest level of overall disability in the sample [71 (s.d. = 20.03)] as well as in all the sub-domains.

### Sensitivity to change

Differences in pre- and post-intervention scores were computed using paired sample *t* test. Small but significant differences were observed at post-intervention in global disability score (ES = 0.19), understanding and communication (ES = 0.17), self-care (ES = 0.22) and life activities scores (ES = 0.23), indicating that DD-CDAS is sensitive to small changes in functioning (see Table 6).

**Table 6.** DD-CDAS sensitivity to change analysis (*N* = 246)

Variable	Pre-test		Post-test		*p*	95% CI		ES
	*M*	s.d.	*M*	s.d.		LL	UL	
Global disability	55.54	22.33	51.54	25.21	0.00	1.79	6.20	0.19
Understanding and communication	58.60	24.51	54.28.	26.02	0.00	1.49	7.14	0.17
Getting around	44.22	41.90	42.47	41.74	0.28	−1.48	4.97	
Self-care	65.09	28.10	58.66	30.73	0.00	3.71	9.14	0.22
Getting along	51.19	28.80	49.12	29.54	0.26	−1.56	5.7	
Life activities	71.88	24.49	66.07	28.37	0.00	2.84	6.76	0.23
Social participation	42.23	21.68	38.61	23.42	0.02	−0.46	8.77	

DD-CDAS, Developmental Disorders-Children Disability Assessment Schedule.

*Note*: The post-test data were collected after 6-month pre-test.

## Discussion

The study results provide evidence that the DD-CDAS satisfies many of the criteria for a pragmatic measurement tool and the data obtained from the implementation of DD-CDAS can be used to answer real-world questions to scale-up of programme for children with developmental disorders in low-resource settings. After only 2 days of training, lay health workers were able to administer the DD-CDAS to caregivers of children with DDs. Administration time was relatively short and the instrument showed good psychometric properties in children with developmental disorders, including high reliability, responsive to the functional disability of children with different developmental difficulties and adequate sensitivity to change over time following treatment.

To rigorously evaluate the factor structure of DD-CDAS, a combination of fit indices was used. Most items fitted in their theoretically assigned domains. The CFA of DD-CDAS showed a rigorous association between items and domains of DD-CDAS. Although each domain of DD-CDAS is unidimensional, given the correlations between domains, they produced a global disability factor. Only one item in the ‘participation in society’ domain exhibited low factor loading and is recommended to be removed from the final version of the tool. Overall, the results indicated that DD-CDAS domains were able to capture disability across the ICF-CY domains and across the various age and developmental conditions-related sub-groups.

While the findings of this study need to be replicated in other settings, the study has several implications for the field of global mental health. The strong inverse correlation between the parent-rated DD-CDAS and specialist-rated DD-CGAS scores strengthens the argument that functioning can be reliably rated by non-specialists. In addition, the DD-CDAS maps well onto the ICF-CY model of childhood functional disability that emphasizes the role of environment and person–environment interaction in determining functional outcomes for children with developmental disorders. It can thus help guide holistic treatment that targets both a child's condition and the contextual factors that influence functioning (Gladstone *et al*., 2010; Charman and Gotham, 2013). Since the DD-CDAS is open access easy to administer in low-resource settings by non-specialist health care workers and sensitive to change, it has the potential to inform the real-world implementation of truly bio-psycho-social interventions for childhood developmental disorders, at-scale in low-resource community settings.

Our results are remarkably similar to the studies conducted using the adult version of the WHODAS-2.0 across multiple cultural settings (Federici *et al*., 2017) and a WHODAS-Child validation study in rural Rwanda (Scorza *et al*., 2013) that assessed children with mental health problems. In the adaptation of WHODAS-Child developed in Rwanda, the domain structure and overall structure of the tool were similarly preserved compared to the original instrument. We added culturally appropriate examples of local functioning to adapt the individual items in the WHODAS-Child to suit the context. Together, these studies suggest that the overall structure of the WHODAS-Child and DD-CDAS represents common domains of functional disability across mental health and developmental conditions for populations in rural Rwanda and rural Pakistan. This is an important attribute to allow cross-population comparability of the research data to inform global health policy and practice.

Sub-group analysis of DD-CDAS scores, according to three age groups 2–5, 6–8 and 9–12 years, revealed statistically significant differences in global disability scores and domain scores in getting around and self-care domains. The scores were higher for children in younger age groups (aged 2–5 and 6–8 years) as compared to children 9–12 years old.

The findings of this study also contribute to a wider academic discourse on the role of functioning in ICD-10 and DSM-V. The Axis VI of the Multiaxial System of Classification of Child and Adolescent Psychiatric Disorders in ICD-10 recommends the use of ‘Global Assessment of Psychosocial Disability’ (GAPD). The tool has been criticized due to insufficient reliability in clinical settings, lack of precision, inability to detect change and limited evidence of concurrent validity (Schorre and Vandvik, 2004). To address this critique, there is an initiative from the WHO to recommend the use of WHODAS-2.0 along with the ICD-11 diagnostic categories (Selb *et al*., 2015). For adults, the American Psychological Association (APA) now suggests that clinicians use the WHODAS-2.0 as a measure of disability (Konecky *et al*., 2014). It is likely that childhood conditions in these classification systems will follow the suit (Glorisa *et al*., 2013). The findings from this study will support these moves, and contribute to moving the field of measurement forward in a substantial way.

### Limitations

These results need to be interpreted with caution since the DD-CDAS was not specifically adapted for the youngest age group (2–5 years) and this sub-sample was not powered to detect statistically significant differences between age groups (though one was found). Further studies with clinical evaluation of the study sample for each of the 2–5, 6–8 and 9–12 age groups are recommended. The sensitivity to change data of DD-CDAS needs to be interpreted with caution as there was no control group for the intervention part of this study and neither did we use any additional measure to assess whether the change at 6 months after pre-test was real and meaningful. Also as the observed effect sizes are small, any RCT will need a larger sample size if DD-CDAS is used as the primary outcome measure. To enhance its utility in clinical settings, further studies with a clinician-administered version of DD-CDAS are needed. Comparison of caregiver- and clinician-rated DD-CDAS can provide useful insights to develop management plans for children with developmental disorders.

## Conclusion

This research shows that DD-CDAS has the potential to be an easy to administer, reliable and valid tool to measure functional disability in children with developmental disorders in low-resource settings. It has the potential to be integrated into global child health programmes to provide data on an individual level, as well as to provide data for comparison across programmes, developmental conditions, cultures and delivery systems in a sustainable and scalable manner. However, further studies are warranted to assess psychometric properties of the tool in different age groups, contexts and for different developmental conditions to validate these findings, and also further evaluate its sensitivity to change over a longer period of follow-up and compared to a typically developing child.

## Footnotes

1 https://opendatakit.org/

† The notes appear after the main text.

## Supplementary material

For supplementary material accompanying this paper visit http://dx.doi.org/10.1017/gmh.2020.10. click here to view supplementary material
