# Supplementary material for: Measuring functional disability in children with developmental disorders in low-resource settings: validation of Developmental Disorders-Children Disability Assessment Schedule (DD-CDAS) in rural Pakistan
Source: Glob Ment Health (Camb). 2020 Jul 13;7:e17. doi: 10.1017/gmh.2020.10 (PMC7443609; doi:10.1017/gmh.2020.10)
Supplement: Supplementary file 1 [file S2054425120000102sup001.docx]

**Additional file A**

**Mapping 36 items of Disability Assessment Schedule for children with Neurodevelopmental Disorders (DAS-NDDs) with the corresponding International Classification of Functioning, Disability and Health, Child and Youth version (ICF-CY) codes ^a^**

| **WHODAS-NDD** | **Domain** | **Domain question** | **ICF-CY Codes** |
| --- | --- | --- | --- |
| **1: Understanding and Communication** | | **In the last 30 days, how much difficulty did your child have in:** |  |
| 1 |  | During the past month, how much difficulty did your child face in concentrating on doing something for ten minutes? (For example while doing school/homework or while playing a game)? | d160 Intentionally focusing on specific stimuli, such as by filtering out distracting noises;  b140 Attention functions (specific mental functions of focusing on an external stimulus or internal  experience for the required period of time);  d110-d129 purposeful sensory experiences |
| 2 |  | During the past month how much difficulty did your child have in remembering to do important things (for example taking along his/her books to school, doing homework on time and remembering to perform daily activities)? | b 144 Memory functions (specific mental functions of registering and storing information and retrieving it  as needed) |
| 3 |  | During the past month how much difficulty did your child come across in analysing and finding solutions to problems in day-to-day life (for example in finding a lost object at school/home)? | d175 Solving problems (finding solutions to questions or situations by identifying and analysing issues, developing options and solutions, evaluating potential effects of solutions, and executing a chosen solution such as in resolving a dispute between two people);  d130-d159 Basic learning |
| 4 |  | During the past month, how much difficulty did your child face in learning a new task (for example while learning in playing a new game, in learning any new activity at school, in learning a new device or mobile)? | d 1551 Acquiring complex skills (Learning integrated sets of actions so as to follow rules and to sequence and coordinate one’s movements, such as learning to play games (e.g. football or chess) and to use a building tool) |
| 5 |  | During the past month, how much difficulty did your child have in generally understanding what people say? | d 310 Communicating with - receiving - spoken messages (comprehending literal and implied meanings of messages in spoken language, such as understanding that a statement asserts a fact or is an idiomatic expression, such as responding and comprehending spoken messages) |
| 6 |  | During the past month, how much difficulty did your child face in initiating and maintaining a conversation with friends and family members? (e.g.; what he/she did? Where did he/she go? Whom did he/she meet)? | d 3550 Discussion with one person (Initiating, maintaining, shaping or terminating an argument or debate  with one person);  d 3501 Sustaining a conversation  (Continuing an interchange by taking turns in vocalizing, speaking or  using sign or shaping a dialogue by adding ideas, introducing a new topic  or retrieving a topic that has been previously mentioned) |
| **2: Getting Around** | | **In the last 30 days, how much difficulty did your child have in:** |  |
| 7 | 2.1. | During the past month, how much difficulty did your child face in standing for a reasonable period of time (for example at social gatherings, at birthdays or wedding functions, at mosques *or while praying,* or in school assembly)? | d 4154 Maintaining a standing position (staying in a standing position for some time as required, such as when standing in a queue. |
| 8 | 2.2. | During the past month, how much difficulty did your child face in standing up from sitting position? | d 4104 Standing (getting into and out of a standing position or changing body position from standing to any other position, such as lying down or sitting down) |
| 9 | 2.3. | During the past month, how much difficulty did your child face in moving around inside their home. (for example in moving around rooms and courtyard)? | d 4600 Moving around within the home (walking and moving around in one’s home, within a room, between rooms, and around the whole residence or living area.) |
| 10 | 2.4. | During the past month, how much difficulty did your child face in moving around at neighbours, friends or relatives’ home? | d 4602 Moving around outside the home and other buildings (walking and moving around close to or far from one’s home and other buildings, without the use of transportation, public or private, such as walking for short or long distances around a town or village.) |
| 11 | 2.5. | During the past month, how much difficulty did you child face in walking a distance which other children of his/her age could easily cover? | d 4501 Walking long distances (walking for more than a kilometre, such as across a village or town, between villages or across open areas) |
| **3: Self-care** | | **In the last 30 days, how much difficulty did your child have in:** |  |
| 12 | 3.1. | During the past month, how much difficulty did your child face in doing following activities by his/herself; In keeping his/ her clothes clean, in taking showers, in cleaning teeth? | d 5101 Washing whole body (applying water, soap and other substances to the whole body in order to clean oneself, such as taking a bath or shower). |
| 13 | 3.2. | During the past month, how much difficulty did your child face in getting him/herself dressed? | d 540 Dressing  (Carrying out the coordinated actions and tasks of putting on and taking off clothes and footwear in sequence and in keeping with climatic and social conditions, such  as by putting on, adjusting and removing shirts, skirts, blouses, pants, undergarments, saris, kimono, tights, hats, gloves, coats, shoes, boots, sandals and slippers) |
| 14 | 3.3. | During the past month, how much difficulty did your child face in having meals by his/herself? | d 550 Eating  (Indicating need for, and carrying out the coordinated tasks and actions of eating food that has been served, bringing it to the mouth and consuming it in culturally acceptable ways, cutting or breaking food into pieces, opening bottles and cans, using eating implements, having meals, feasting or dining) |
| 15 | 3.4. | During the past month, how much difficulty did your child face in keeping himself /herself safe in the absence of elders (for example from hot and sharp objects, from electric switch boards, from strangers in the neighbourhood? | d510-d650 Combination of multiple self-care and domestic life tasks |
| **4: Getting along with people** | | **In the last 30 days, how much difficulty did your child have in:** |  |
| 16 | 4.1. | During the past month, how much difficulty did your child face in getting along with people who are not known or familiar, (for example distant relatives)? | d 730 Relating with strangers  (Engaging in temporary contacts and links with strangers for specific purposes, such  as when asking for information, directions or making a purchase.) |
| 17 | 4.2. | During the past month, how much difficulty did your child face in maintaining friendly relations with other children of his/her age? | d 7500 Informal relationships with friends (Creating and maintaining friendship relationships that are characterized by mutual esteem and common interests.) |
| 18 | 4.3. | During the past month, how much difficulty did your child face in maintaining friendly relations with his/her siblings and family members? | d 760 Family relationships  (creating and maintaining kinship relationships, such as with members of the nuclear family, extended family, foster and adopted family and step-relationships,  more distant relationships such as second cousins, or legal guardians.)  d 750 Informal social relationships  (entering into relationships with others, such as casual relationships with people  living in the same community or residence, or with co-workers, students, playmates or people with similar backgrounds or professions.) |
| 19 | 4.4. | During the past month, how much difficulty did your child face in making new friends? | d 7500 Informal relationships with friends (creating and maintaining friendship relationships that are characterized  by mutual esteem and common interests.)  d 7200 Forming relationships  (beginning and maintaining interactions with others for a short or long period of time, in a contextually and socially appropriate manner, such as by introducing oneself, finding and establishing friendships and  professional relationships, starting a relationship that may become  permanent, romantic or intimate.) |
| 20 | 4a* | During the past month, how much difficulty did your child face in getting along with people who are not a part of your family e.g. neighbours, teachers, friends and distant relatives? | d 7200 Forming relationships  (beginning and maintaining interactions with others for a short or long period of time, in a contextually and socially appropriate manner, such  as by introducing oneself, finding and establishing friendships and  professional relationships, starting a relationship that may become  permanent, romantic or intimate.) |
| **5: Life activities** | | **In the last 30 days, how much difficulty did your child have in:** |  |
| 21 | 5.1. | In the past month, how much difficulty did your child face in helping you with household activities which are expected from other children of his/her age? | d6 Domestic life |
| 22 | 5.2. | During the past month, how much difficulty did your child face in doing well the chores he/she was supposed to do? | d640 Doing housework; d210 Undertaking a single task;  d220 Undertaking multiple tasks |
| 23 | 5.3. | During the past month, how much difficulty did your child face in completing the assigned tasks/chores? | d640 Doing housework; d210 Undertaking a single task; d220 Undertaking multiple tasks |
| 24 | 5.4. | During the past month, how much difficulty did your child face in getting the household errands quickly when it is important? | d640 Doing housework; d210 Undertaking a single task;  d220 Undertaking multiple tasks |
| 24 | 5.5. | During the past month, how much difficulty did your child face in doing his or her day-to-day school work (for example school and homework) | 830 Higher education; d825 Vocational training; d820 School education |
| 25 | 5.6. | During the past month, how much difficulty did your child have in preparing well for his/her important test or exam? | d830 Higher education; d825 Vocational training; d820 School education; d210 Undertaking a single task; d 220 Undertaking multiple tasks |
| 27 | 5.7. | During the past month how much difficulty did your child have in completing the given homework? | d830 Higher education; d825 Vocational training; d820 School education; d 210 Undertaking a single task; d220 Undertaking multiple tasks |
| 28 | 5.8. | During the past month, how much difficulty did your child have in completing the homework as quickly as needed? | d830 Higher education; d825 Vocational training; d820 School education; d210 Undertaking a single task; d220 Undertaking multiple tasks |
| 29 | 5a* | During the past month, how much difficulty did your child face in following school’s rules & regulations and in getting familiar with school’s environment? | d 720 Complex interpersonal interactions  (Maintaining and managing interactions with other people, in a contextually and  socially appropriate manner, such as by regulating emotions and impulses,  controlling verbal and physical aggression, acting independently in social interactions, and acting in accordance with social rules and conventions.)  d 820 School education  (Gaining admission to school, education; engaging in all school-related responsibilities and privileges; learning the course material, subjects and other curriculum requirements in a primary or secondary education programme, including attending school regularly; working cooperatively with other students, taking direction from teachers, organizing, studying and completing assigned tasks and projects, and advancing to other stages of education.) |
| **6: Participation in society** | | **In the last 30 days, how much difficulty did your child have in:** |  |
| 30 | 6.1. | During the past month, how much difficulty did your child have in joining social activities (like wedding functions, school fairs, religious gatherings, after school activities, extra-curricular activities)? | d910 Community life |
| 31 | 6.2. | In your opinion, to what extent your child did not get the opportunities to take part in social activities due to barriers and hindrances around him/her the way they ought to have been given to him/her? | d9 Community, social and civic life |
| 32 | 6a* | During the past month, how much of your time was spent in taking care of your child’s health conditions and other problems related to it? | Not applicable (impact question) |
| 33 | 6.5. | During the past month, how much has your child been emotionally affected by his/her health problems? / issues? | b 152 Emotional functions  (specific mental functions related to the feeling and affective components of the  processes of the mind.) |
| 34 | 6.6. | During the past month, how far your child’s health issues acted as a drain on your financial resources? | d8700 Personal economic resources (Having command over personal or private economic resources, in order  to ensure economic security for present and future needs.) |
| 35 | 6.7 | During the past month, how much of a problem did you or the rest of your family have because of your child’s health problems? | Not applicable (impact question) |
| 36 | 6.8. | During the past month, how much difficulty did your child face in doing things that would keep him pleased/ mentally relaxed; (activities like recitation of Quran and naat, doing exercise, watching TV and playing video games)? | d 920 Recreation and leisure |
